# Popular Pastimes as Aids to Hospital Funds

**Published:** 1890-01-25

**Authors:** A. W. Webster

**Affiliations:** An Assistant Secretary to the London Hospital Saturday Fund


### Popular Pastimes as Aids to Hospital Funds.

By A. W. Webster, An Assistant Secretary to the London Hospital Saturday Fund.

*(Continued from page 237.)*

Demonstrations.

Trade and benefit society demonstrations in aid of charities are recognised annual events in many towns, though the practice is by no means universal. London is probably the only city where charity demonstrations are organised indiscriminately (and consequently overdone in some localities), and it certainly excels all provincial towns in the gorgeousness of its processions of this nature, though I am doubtful if it will compare very favourably in the more important item of money raised.

The double object of aiding the hospitals and advertising the societies is attained, and, I think, aimed at by most who take part in these displays, and we have no reason of complaint that it is so, for both hospitals and societies are identical in their desire to alleviate distress, and the one object is, therefore, almost as commendable as the other.

The more successful of these demonstrations are carefully organised on something like the following lines. Delegates from each friendly and trade society in the town and district are invited to meet and form themselves into a central committee of management.

In planning the routes care is taken to parade the principal thoroughfares with the full strength of the procession. Detachments are planned to traverse cross and intervening streets and timed to join the main ranks at certain prearranged localities. Stewards are appointed in connection with every society represented, and these officials walk on the outside of the ranks with collecting-boxes for the purpose of gathering subscriptions from the passers-by. The processionists, and any of the public who choose to follow, are then massed in the largest available buildings, and addresses suitable to the occasion are delivered by popular public speakers, the proceedings terminating with collections in aid of the charities.

Many of these demonstrations are now carried out on Sundays, churches and chapels being used for meeting places. Some arrange with popular clergymen to conduct the services, but others prefer to have the assistance of experienced laymen. The churches and chapels are usually obtainable free of cost, either on Sundays or week days, for such gatherings when timely application is made to the proper authorities.

The friendly society demonstration at Sunderland is held on a Sunday afternoon, the processionists using the public park as a rendezvous, and after parading the town, mass themselves in the theatres, the gratuitous use of which is granted by the proprietors. These buildings can also be got for Saturday afternoon demonstrations almost as readily as for those conducted on Sundays, and where any difficulty is experienced in securing a sufficient number of speakers they possess the advantage of greater size over other buildings, and enable the committee more easily to centralise their members.

Brass bands are necessary accompaniments to all successful processions. As it often happens that some of the societies interested in the arrangements have such bodies connected with themselves, no great difficulty is experienced in providing this part of the display.

Some societies, wishful to provide a more impressive spectacle than their neighbours, occasionally engage the services of a band to head their detachment, and pay the cost from their own funds. Other spectacular features of the demonstration are also dealt with privately by individual societies, each defraying its own expenses.

Individual societies, strong in membership, and with many separate lodges within a given area, sometimes desire to arrange and carry out charity demonstrations themselves. As the danger is rather to underdo than overdo schemes for hospital support, I see no reason why these should not be encouraged and supported, provided ordinary care be used to prevent the public from becoming surfeited with a series of weak and ineffective efforts, and that the private displays be kept at a sufficiently remote date from the general combined demonstration not to materially interfere with its operations.

Torchlight processions have occasionally been carried out with great success, and in districts where a change from the ordinary style is desired this might prove acceptable.

